# Circular RNA VMA21 ameliorates sepsis‐associated acute kidney injury by regulating miR‐9‐3p/SMG1/inflammation axis and oxidative stress

**DOI:** 10.1111/jcmm.15741

**Published:** 2020-08-22

**Authors:** Yan Shi, Chuan‐Fu Sun, Wen‐Han Ge, Ye‐Ping Du, Nan‐Bin Hu

**Affiliations:** ^1^ Department of Emergency The Affiliated Huai’an Hospital of Xuzhou Medical University and The Second People’s Hospital of Huai’an Huai’an China; ^2^ Department of Intensive Care Unit Lian Shui People’s Hospital Lian Shui Jiangsu China

**Keywords:** circVMA21, inflammation, miR‐9‐3p, oxidative stress, SMG1

## Abstract

Accumulating evidence suggests that circular RNAs have the abilities to regulate gene expression during the progression of sepsis‐associated acute kidney injury. Circular RNA VMA21 (circVMA21), a recent identified circular RNA, could reduce apoptosis to alleviate intervertebral disc degeneration in rats and protect WI‐38 cells from lipopolysaccharide‐induced injury. However, the role of circVMA21 in sepsis‐associated acute kidney injury (sepsis‐associated AKI) is unknown. In this study, we first demonstrated that circVMA21 alleviated sepsis‐associated AKI by reducing apoptosis and inflammation in rats and HK‐2 cells. Additionally, to explore the molecule mechanism underlying the amelioration, after the bioinformatics analysis, we confirmed that miR‐9‐3p directly bound to circVMA21 by luciferase and RNA immunoprecipitation assay, and the effector protein of miR‐9‐3p was SMG1. Furthermore, the oxidative stress caused by sepsis‐associated AKI was down‐regulated by circVMA21. In conclusion, circVMA21 plays an important role in the regulating sepsis‐associated AKI via adjusting miR‐9‐39/SMG1/inflammation axis and oxidative stress.

## 1 INTRODUCTION

Sepsis is a severe, rapid develop disease with progressive organ failure caused by host's response to infections, which leads to a high mortality.^1^ Also, sepsis is considered as a predisposing factor for acute kidney injury, for the most often affected organ in sepsis is the kidney.^2^, ^3^, ^4^, ^5^ Sepsis‐associated acute kidney injury (sepsis‐associated AKI) causes huge medical care burden for the society; however, the pathophysiologic mechanisms of sepsis‐associated AKI remain largely unknown.

Non‐protein‐coding RNAs or more simply non‐coding RNAs are important molecules regulating gene expression during development and disease, and the dysregulation of non‐coding RNAs generally associated with the abnormalities of the body.^6^ Circular RNAs (circRNAs), which are enriched in the kidney, can regulated the gene expression by acting as a sponge for RNAs or scaffolds for transcription factors. CircRNAs play essential roles in multiple renal diseases as the regulators of gene expression.^7^, ^8^ The expression pattern of circRNAs is changed in the kidney of a mice AKI model, indicating that circRNAs may participate in maintaining the renal homeostasis.^9^ Circular RNA VMA21 (circVMA21, hsa_circ_0091702 in CircBase), deriving from vacuolar ATPase assembly factor, is first identified as a regulator to alleviate intervertebral disc degeneration (IVDD) via targeting miR‐200c and X linked inhibitor‐of‐apoptosis protein.^10^ Also, circVMA21 attenuates the lipopolysaccharide‐induced inflammatory damages through targeting miR‐142‐3p in WI‐38 cells.^11^ In this study, we explored the function of circVMA21 in the sepsis‐associated AKI, which was also accompanied by apoptosis and inflammatory injuries in the kidney, using both LPS‐treated HK‐2 cells and caecal ligation and puncture (CLP)‐induced rat sepsis‐associated AKI model.

Here, we found that circVMA21 could alleviate sepsis‐associated AKI symptoms by evaluating the levels of general biomarkers of AKI. Next, we confirmed that circVMA21 reduced apoptosis and inflammation in both rat model and HK‐2 cells. Furthermore, we identified miR‐3‐9 as the direct target of circVMA21, and SMG1 as the effector protein in circVMA21/miR‐9‐3p axis. Also, circVMA21 could reduce the oxidation stress in the development of sepsis‐associated AKI. In summary, we found circVMA21 alleviated sepsis‐associated AKI via regulating miR‐9‐3p/SMG1/inflammation and oxidative stress.

## 2 MATERIALS AND METHODS

### 2.1 Animals and the establishment of sepsis‐associated AKI model

Wistar rats purchased from SLAC Laboratory Animal Co., Ltd, China, were all housed at 22°C and regular light‐dark cycle (12:12). Caecal ligation and puncture in rats was applied to mimics the sepsis‐associated AKI clinical symptoms.^12^ For the rats in Sham group, the caecum was exposed without perforation, and for the rats in CLP group, the caecum was exposed, ligated and then perforated. After surgery, rats were placed in a warm environment to recover‐, and monitored before the following experimental procedures.

### 2.2 Injection of circ‐VMA21

To recapitulate circ‐VMA21, the third exon of VMA21 with 1 kb flanking introns was amplified and subcloned into the pcDNA3.1 vector. After the recovery of the CLP surgery, rats were anaesthetized with injection of katemine and xylazine. Around 30ul of adeno‐associated virus containing the circ‐VMA21 cassette or pcDNA3.1 vectors was delivered into 3 different sites of the renal tissue using a 33‐gauge needle.

### 2.3 Haematoxylin and eosin (HE) staining and the evaluation of kidney morphology

Kidney tissues were embedded with paraffin and then sectioned into 5 μm slices. The kidney sections were stained with stained haematoxylin‐eosin (HE) (Solarbio Science & Technology Co., Ltd, Beijing, China). We then determined the degree of kidney injury based on the morphology changes observed under a light microscope. The scoring criteria from 0 to 5 as follows: 0 = normal morphology; 1 = degeneration only without necrosis; and 2 (<25%), 3 (<50%), 4 (<75%), and 5 (>75%) = necrosis, vacuolar degeneration, tubular dilatation and haemorrhage.^13^

### 2.4 Measurement of BUN, sCr, uKim‐1, uNGAL in the kidney of rats

Blood samples were collected from the rats and centrifuged at 5000 *g* for the collection of the serum. Levels of blood urea nitrogen (BUN) and serum creatinine (sCr) were measured with BUN detection kit and serum creatinine detection kit (StressNarq Bioscience, British Columbia, Canada). Urine samples were collected and centrifuged at 600 *g* for 5 minutes and used for the measurement of the levels of urine NGAL and KIM‐1 with the ELISA kit (Cusabio Biotech, Zhengzhou, China) following the instructions.

### 2.5 Culture and bacterial LPS treatment of HK

Human kidney 2 (HK‐2) cells were obtained from the Cell Bank of the Chinese Academy of Sciences, and cultured with Minimum Essential Medium (Gibco, Agawam, MA, USA) containing 5 ng/mL human recombinant epidermal growth factor (EGF) (Novus, Centennial, CO, USA) and 10% foetal bovine serum (Gibco). To model sepsis‐associated AKI, HK‐2 cells were exposed to 10 μg/mL LPS (Sigma, Darmstadt, Germany).

### 2.6 Apoptosis examination

HK‐2 cells were seeded at a density of 4.0 × 10^5^ per well. After the treatment of LPS, HK‐2 cells were harvested and suspended in binding buffer. Then, Annexin V/PI kits (KeyGEN, Nanjing, China) were applied for the detection of apoptosis. Flow cytometric analysis for the apoptotic rate were conducted using a FACSAria flow cytometer (BD Biosciences, San Jose, CA, USA). To detect the apoptosis levels of renal tissues, we applied the TUNEL assay. To quantify the apoptosis level of tissues, we analyse the brown TUNEL positive cells per field in each group.

### 2.7 Total RNA extraction and qRT‐PCR

Total RNA was extracted with TRIzol reagent (Invitrogen, Agawam, MA, USA) following the manufacturer's instructions. After quality measurement of total RNA using a NanoDrop 2000 (Thermo Scientific, Agawam, MA, USA), cDNA was synthesized using PrimeScript RT reagent kit (Takara, Beijing, China). SYBR Premix Ex Taq II (Takara, Beijing, China) and a LightCycler 96 System (Roche, Basel, Switzerland) were used. For the quantification of the expression of the genes, the relative quantification method, 2‐ΔΔCT, was employed. Primers for the amplification of GAPDH, Bax, Bcl‐2, Active caspase3, TNF‐α, IL‐6 and IL‐1β were as follows:

GAPDH: Forward: 5'‐TTCAATGGCACAGTCAAGGC‐3', Reverse: 5'‐TCACCCCATTTGATGTTAGCG‐3'; Bax: Forward: 5'‐ATGGAGCTGCAGAGGATGA‐3', Reverse: 5'‐CCAGTTTGCTAGCAAAGTAG‐3'; Bcl‐2: Forward: 5'‐GAGGATTGTGGCCTTCTTTG‐3', Reverse: 5'‐AGGTACTCAGTCATCCACA‐3'; Caspase 3: Forward: 5'‐GCTGGACTGCGGTATTGAGA‐3', Reverse: 5'‐CCATGACCCGTCCCTTGA‐3'; TNF‐α: Forward: 5'‐CTCCCAGAAAAGCAAGCAAC‐3', Reverse: 5'‐CGAGCAGGAATGAGAAGAGG‐3'; IL‐6: Forward: 5'‐GGATACCACCCACCACAGACCAG‐3', Reverse: 5'‐CGATGAGTTTTCTGACAGTGCATCATC‐3'; IL‐1β: Forward: 5'‐CTGTGACTCGTGGGATGATG‐3', Reverse: 5'‐GGGATTTTGTCGTTGCTTGT‐3'.

### 2.8 Western blotting

Total protein of both HK‐2 cells and rat renal tissues were prepared with protein extraction kit (Beyotime Biotech. Co., Ltd, Shanghai, China). After measuring the protein concentrations using the BCA method, total protein samples were subjected SDS‐PAGE and then transferred to the PVDF membrane. The PVDF membrane was blocked with PBST containing 5% BSA, washed for three times with PBST and incubated in the primary antibody solution overnight at 4°C. Then, the membrane was washed for three time and incubated in the secondary antibody solution. The signal of was determined with Pierce ECL Western Blotting Substrate (Thermo Fisher Scientific, Agawam, MA , USA). Antibodies used were as follows: rabbit anti‐GAPDH (Abcam, Cambridge, MA, USA, 1:5000); rabbit anti‐Bax (Abcam, Cambridge, MA, 1:5000); rabbit anti‐Bcl‐2 (Abcam, Cambridge, MA, 1:5000); rabbit anti‐Caspase‐3 (Cell Signaling Technology, Danvers, MA, 1:1000); goat anti‐TNF‐α (R&D Systems, Minneapolis, MN, USA, 1:300); rabbit anti‐IL6 (Boster, San Mateo, CA, USA, 1:500); rabbit anti‐IL‐1β (GeneTex, Irvine, CA, USA, 1:1000).

### 2.9 Luciferase assay

To confirm the direct interaction of circular RNA VMA21 (circVMA21) and miR‐9‐3p, circVMA21 fragment containing wild‐type or mutated miR‐9‐3p binding sites were synthesized and, respectively, cloned into luciferase vectors. circVMA21 reporter plasmids were cotransfected with miR‐9‐3p mimics into HK‐2 cells. And to confirm the direct interaction of miR‐9‐3p and SMG1 mRNA, the miR‐9‐3p reporter for the luciferase assay was constructed. The luciferase activity was measured and analysed using a dual‐luciferase reporter assay system (Promega, Madison, WI, USA) under the manufacturer's instruction.

### 2.10 RIP‐AGO2 and RNA pull down

HK‐2 cells transfected with miR‐9‐3p or miR‐NC mimics were harvested and lysed. And the supernatant was collected after centrifugation. Anti‐AGO antibodies (Abcam) or control IgG were used for the RNA immunoprecipitation assay. Followed by RT‐qPCR test for the expression level of circVMA21, the direct binding of circVMA and miR‐9‐3p was confirmed.

### 2.11 Terminal deoxynucleotidyl transferase dUTP nick end labelling (TUNEL) assay

2 × 10^6^ HK‐2 cells were collected and incubated with 4% paraformaldehyde (PFA) at room temperature for fixation. Then, the fixed cells were incubated with 0.2% Triton X‐100, and then in FITC labelled dUTP and terminal deoxynucleotidyl transferase. After imaging with an epifluorescence microscope at 400× magnification, the apoptosis degree was determined by the number of TUNEL positive cells.

### 2.12 RNA interference for the down‐regulation of SMG1

The siRNA targeted SMG1 specially and its corresponding random control sequence were synthesized from Genewiz Co., Ltd, Suzhou, China, and then subcloned into the pSuper vector, which could be transfected into cells to down‐regulate SMG1.

### 2.13 Determination of oxidative stress parameters

The levels of reactive oxygen species (ROS), malondialdehyde (MDA) and glutathione (GSH) in the rat kidney were measured with Reactive Oxygen Species Assay Kit (Beyotime), Lipid Peroxidation MDA Assay Kit (Beyotime) and Total Glutathione Assay Kit (Beyotime), respectively. The activities of superoxide dismutase (SOD) were determined using Total Superoxide Dismutase Assay Kit with WST‐8 (Beyotime), and kidney catalase (CAT) activity was determined based on H_2_O_2_ consumption, following the procedure established by Aebi in 1984.^14^

### 2.14 Statistical analysis

Data were presented as mean ± SEM and compared by Student's *t* test, and *P* < 0.05 was considered statistically significant. All data were analysed using Graphpad Prism 7.0 (San Diego, CA, USA).

## 3 RESULTS

### 3.1 circVMA21 alleviated the symptoms of CLP‐induced sepsis‐associated AKI rat model

**FIGURE 1:**
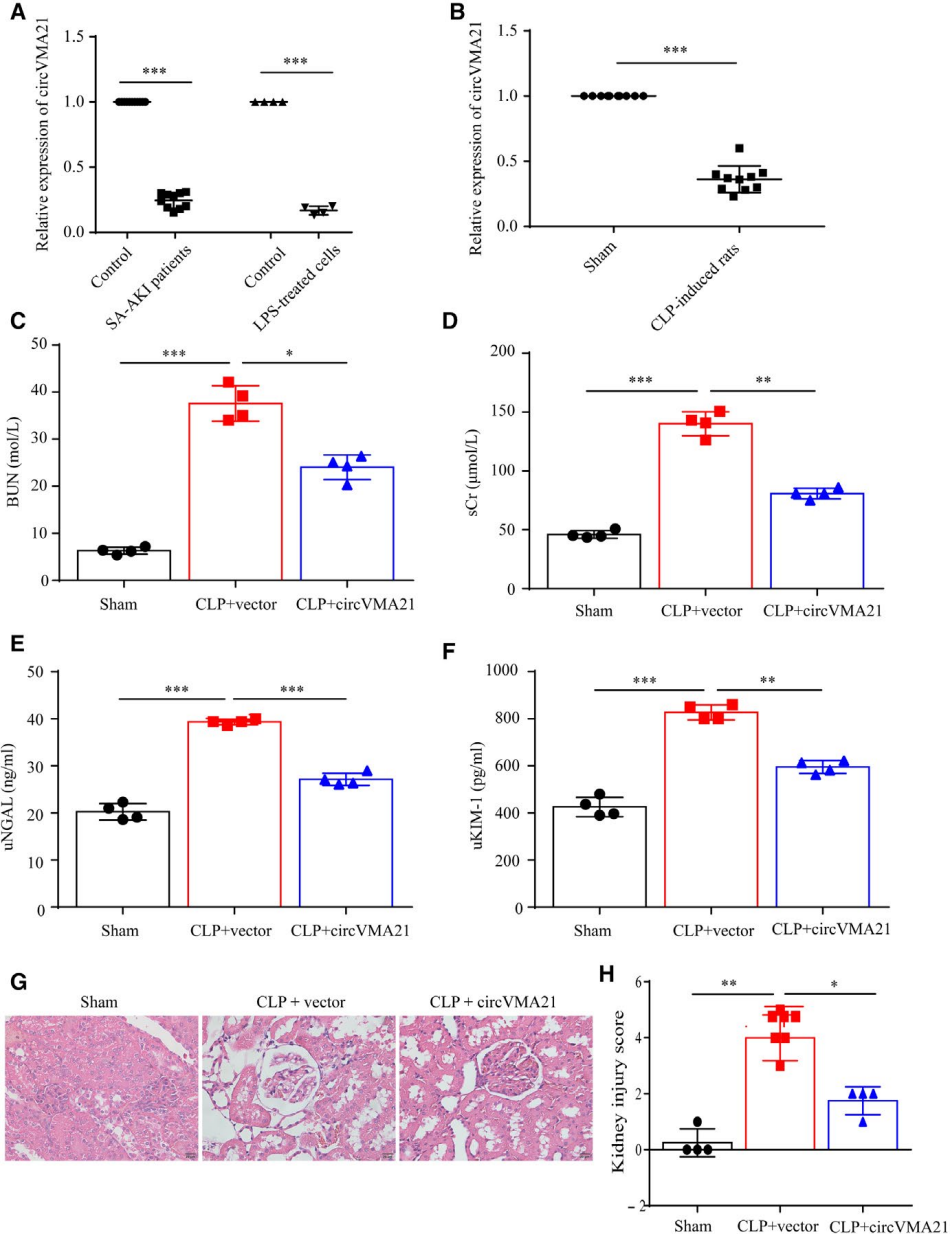
circVMA21 alleviated the symptoms of CLP‐induced sepsis‐associated AKI rat model. (A) Relative expression level of circVMA21 in sepsis‐associated AKI patients and LPS‐treated HK‐2 cells. (B) Relative expression level of circVMA21 in CLP‐induced AKI rats. (C) Blood urea nitrogen (BUN) and (D) serum creatinine (sCr) levels in rat blood samples. (E) Urine neutrophil gelatinase‐associated lipocalin (uNGAL) and (F) urine kidney injury molecule‐1 (uKIM‐1) levels in rat urine samples. (G) Pathological morphology HE (haematoxylin‐eosin) staining of the renal tissue. (H) Semi‐quantitative histopathological score of renal tissue on a scale of 0‐5 based on images with a magnification of 400. *P < 0.05, ***P < 0.001. Data were presented as mean ± SD from four independent experiments. Sham: the rat caecum was exposed without perforation; CLP: caecal ligation and puncture (CLP) surgery for Wistar rats

We found in sepsis‐associated acute kidney injury patients, circVMA21 was significantly down‐regulated, and in LPS‐treated HK‐2 cells, the expression level of circVMA21 was also down‐regulated (Figure 1A). To confirm that CLP‐induced rats model mimics the circVMA21 expression phenotype, we collected the rats kidney samples and analysed the expression level of circVMA21 (Figure 1B). We firstly investigated the levels of the general biomarkers of AKI, BUN, sCr, urinary neutrophil gelatinase‐associated lipocalin (uNGAL) and urinary kidney injury molecule‐1 (uKIM‐1), in the Sprague‐Dawley rats exposed to CLP, which modelled the sepsis‐associated AKI. The levels of BUN, sCr, uNGAL and uKIM‐1 were significantly increased in the CLP group compared to that in the sham group, while the degree of sepsis‐associated AKI was markedly alleviated in the CLP rats with overexpressed circVMA21 compared to the CLP group (Figure 1C‐F). To further verify the function of circVMA21 in vivo, the HE staining of the renal tissue from CLP rats overexpressed circVMA21 exhibited less vacuolar degeneration and haemorrhage (Figure 1G). The degree of sepsis‐associated AKI indicated by the semi‐quantitative histopathological score of renal tissue also suggested circVMA21 alleviates the symptoms of sepsis‐associated AKI (Figure 1H).

### 3.2 circVMA21 attenuated sepsis‐associated AKI by decreasing oxidation stress

**FIGURE 2:**
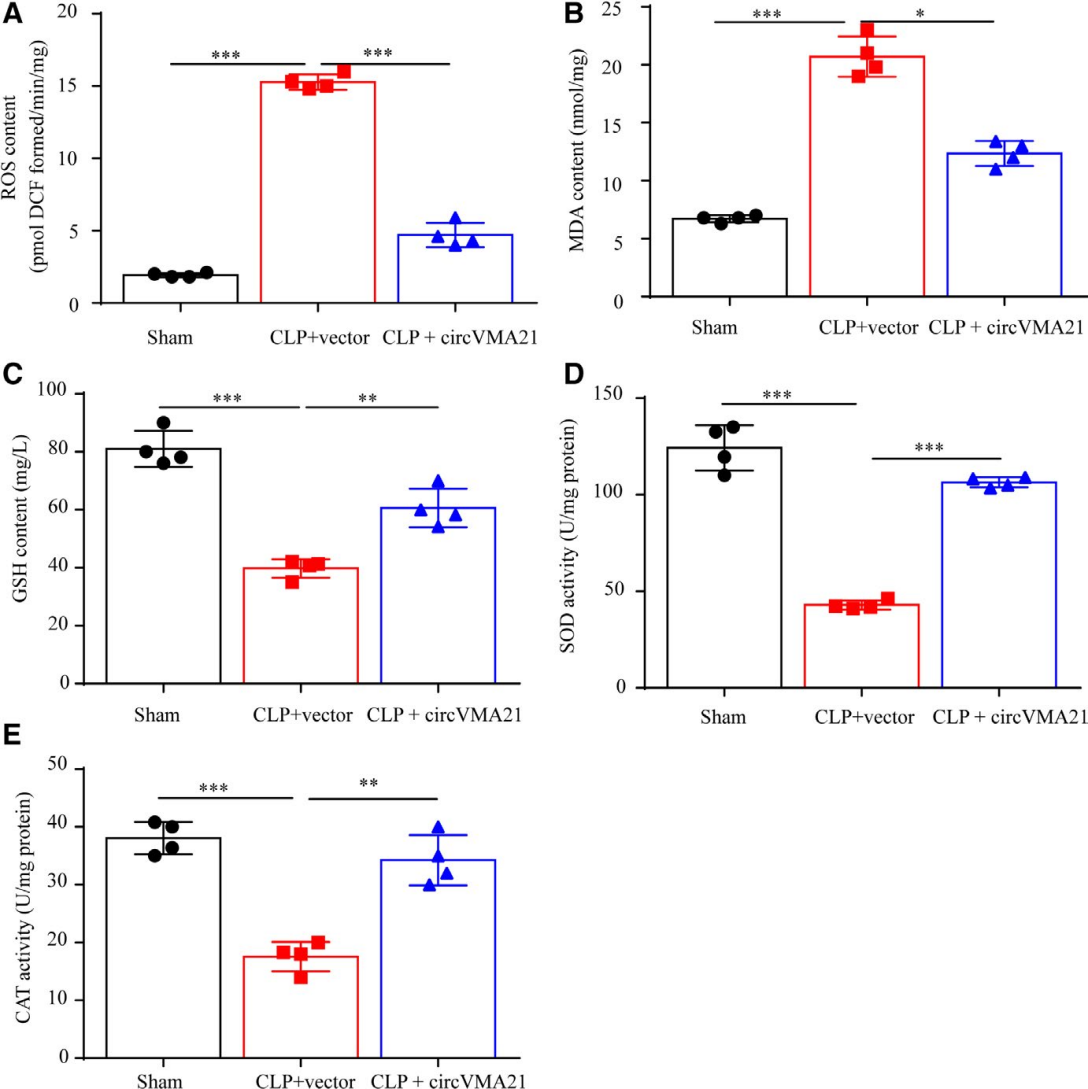
circVMA21 attenuated sepsis‐associated AKI by decreasing oxidation stress. The content of (A) reactive oxygen species (ROS), (B) malondialdehyde (MDA) and (C) glutathione (GSH) in the kidney tissue were measured. The activities of (D) superoxide dismutase (SOD) and (E) catalase (CAT) in the kidney of sepsis‐associated AKI rats were determined. *P < 0.05, ***P < 0.001. Data were presented as mean ± SD from four independent experiments

In the kidney, multiple pathways, which generate excessive ROS, are identified to be correlated with kidney disease.^15^ For instance, smad3, a well‐documented downstream signalling molecules of TGF‐β correlated with chronic kidney disease, could bind to the promoter region of NOX4 and generate excessive ROS and inflammation.^16^ In the development of AKI, the oxidation stress and following oxidative damage were largely involved.^17^ To evaluate the oxidative stress parameters within sepsis‐associated AKI, we measured the content of ROS, malondialdehyde (MDA), glutathione (GSH) and determined the activities of SOD and catalase (CAT) in the kidney of sepsis‐associated AKI rats. We found that sepsis‐associated AKI markedly increased the levels of ROS and MDA, but decreased the level of GSH and the activities of SOD and CAT in the kidney of the rats. By up‐regulation of circVMA21, the oxidative damage caused by sepsis‐associated AKI was notably reduced, indicating that circVMA21 also functioned against oxidation stress to attenuate sepsis‐associated AKI (Figure 2A‐E).

### 3.3 circVMA21 reduced apoptosis and inflammation caused by sepsis‐associated AKI in both LPS‐induced HK‐2 cells and CLP‐induced rats

**FIGURE 3:**
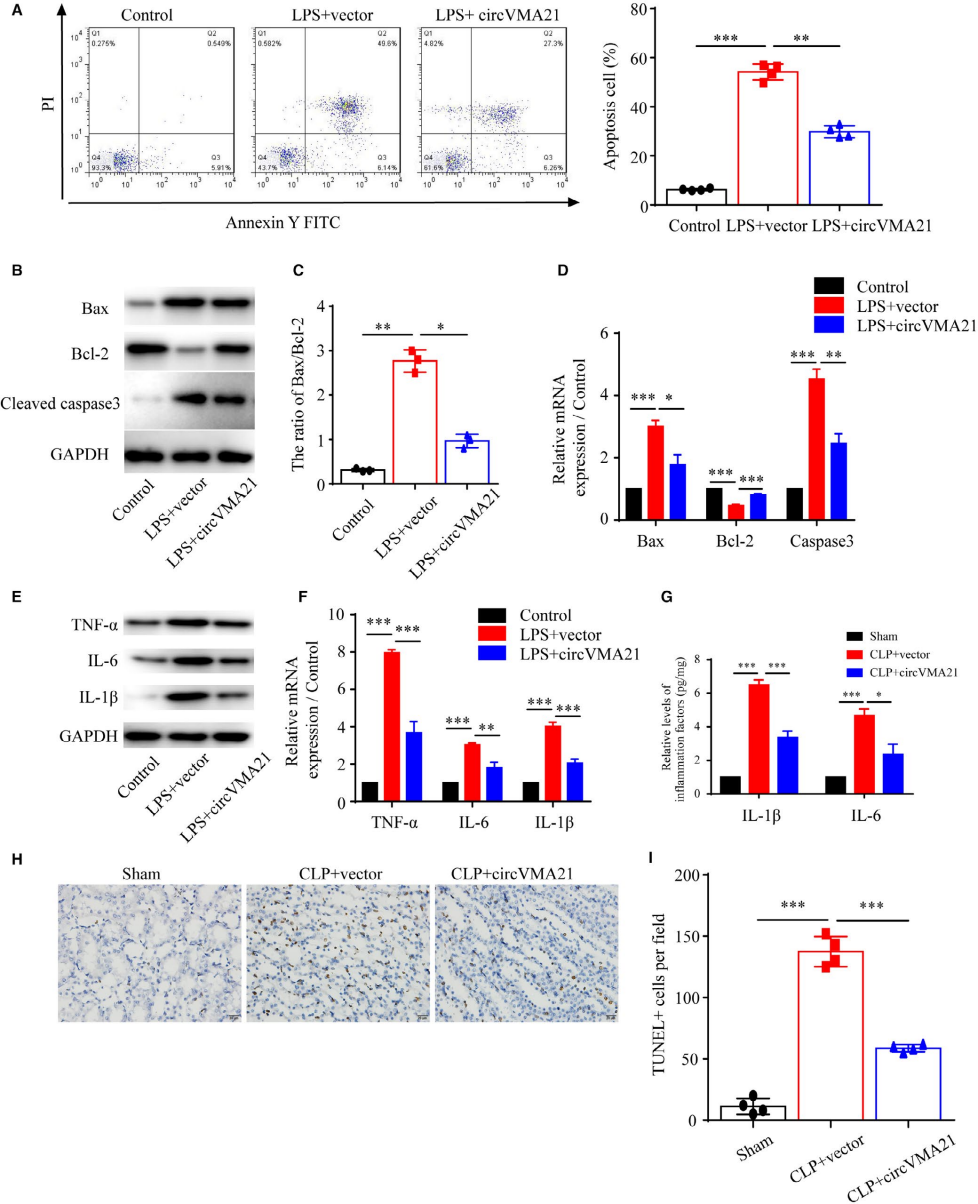
circVMA21 reduced apoptosis and inflammation caused by sepsis‐associated AKI in both LPS‐induced HK‐2 cells and CLP‐induced rats. (A) Apoptosis levels of HK‐2 cells control, cells treated with bacterial LPS or bacterial LPS and overexpression of circVMA21 were measured by flow cytometry. (B) Levels of apoptosis related proteins, Bax, Bcl‐2 and active Caspase3 were measured by western blot (WB). (C) The ratio of Bax/Bcl‐2 was calculated. (D) Relative mRNA expression levels of Bax, Bcl‐2 and Caspase3 were measured with quantitative PCR. (E) Levels of inflammation related proteins, TNF‐α, IL‐6 and IL‐1β, were measured with WB. (F) Relative mRNA expression levels of TNF‐α, IL‐6 and IL‐1β were measured with quantitative PCR. (G) Relative levels of IL‐1β and IL‐6 in CLP‐induced sepsis‐associated AKI rats. (H) Apoptosis of renal tubular epithelial cells was detected using TUNEL method. (I) Quantification of the TUNEL positive apoptosis cells per field. Apoptosis cells nuclei were stained with brown TUNEL positive cells. *P < 0.05, ***P < 0.001. Data were presented as mean ± SD from three independent experiments

Then, the cellular mechanisms underlying the phenotypes that circVMA21 alleviated sepsis‐associated AKI were specifically analysed. Bacterial LPS treatment of HK‐2 cells induced apoptosis, while circVMA21 up‐regulation significantly inhibited apoptosis (Figure 3A). Consistent with the flow cytometry result, we confirmed that circVMA21 inhibited apoptosis induced by LPS by measuring the expression level of Active caspase3 and the ratio of Bax/Bcl‐2 (Figure 3B‐D). We also found that circVMA21 reduced inflammation with the detection of inflammation related proteins, TNF‐α, IL‐6 and IL‐1β, in LPS‐induced HK‐2 cells (Figure 3E,F). Also, the levels of inflammation factors in CLP‐induced rats, IL‐6 and IL‐1β, were reduced by overexpression of circVMA21 (Figure 3G). Also, the apoptosis results of renal tubular epithelial cells obtained from TUNEL staining of the kidney tissue from CLP‐induced sepsis‐associated AKI rats indicated circVMA21 inhibited apoptosis in *vivo*, which was identical to the in *vitro* results (Figure 3H,I).

### 3.4 circVMA21 functioned as a sponge of miR‐9‐3p

**Figure 4:**
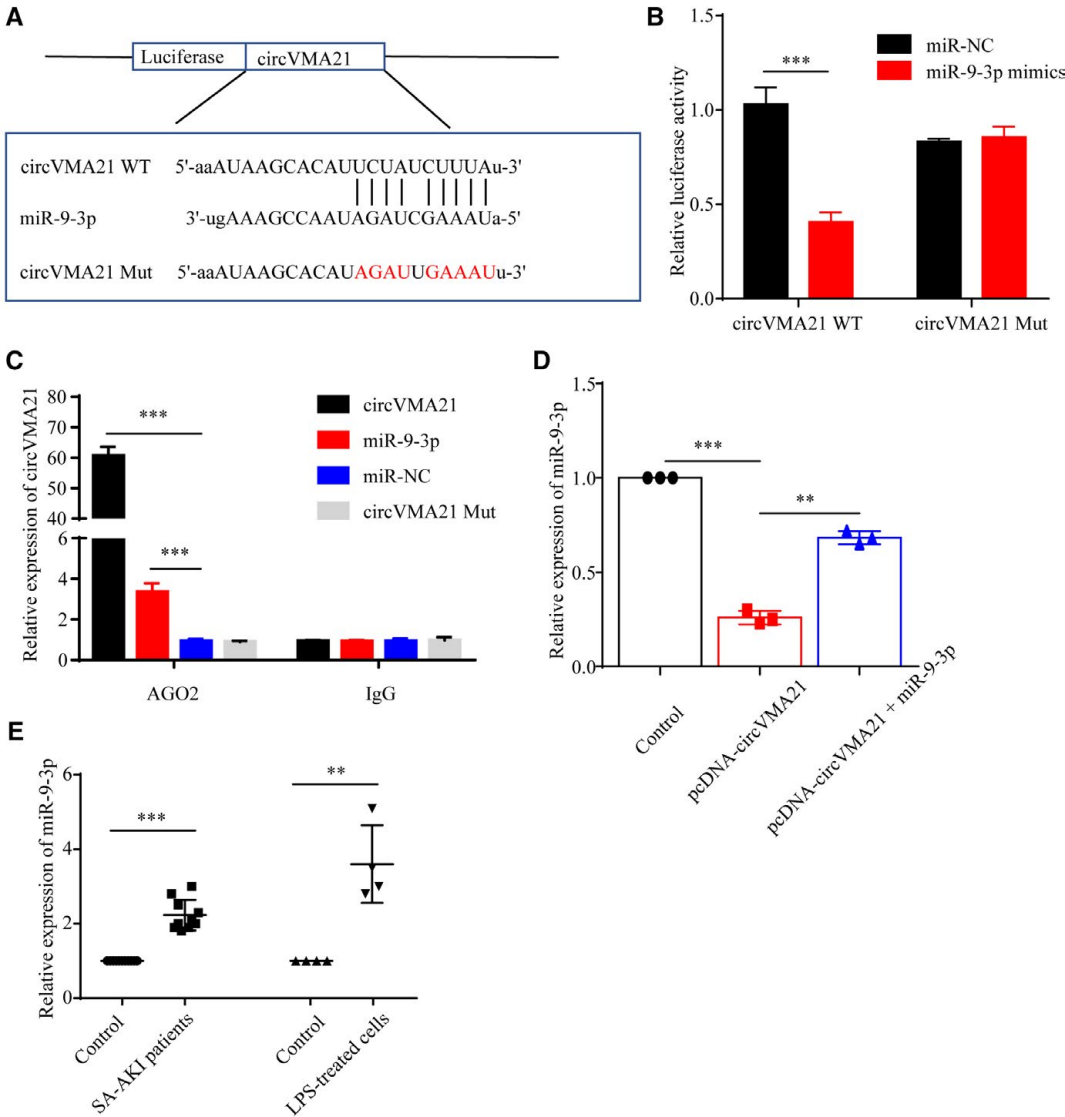
circVMA21 bound directly with miR‐9‐3p. (A) The binding sites between circVMA21 and miR‐9‐3p. And the luciferase reporter designs with the wild‐type and mutant circVMA21. (B) Wild‐type or mutant circVMA21 were cotransfected with miR‐9‐3p, and then, the luciferase activity of circVMA21 reporter vector was measured. (C) RNA immunoprecipitation (RIP) assays were performed using an anti‐AGO2 antibody to verify the correlation between circVMA21 and miR‐9‐3p in HK‐2 cells. (D) Relative miR‐9‐3p expression in control HK‐2 cells and HK‐2 cells transfected with pcDNA‐circVMA21 or pcDNA‐circVMA21 + miR‐9‐3p. (E) Relative expression level of miR‐9‐3p in sepsis‐associated AKI patients and LPS‐treated HK‐2 cells. *P < 0.05, ***P < 0.001. Data were presented as mean ± SD from three independent experiments. IgG: immunoglobulin G

Most of the circular RNAs (circRNAs) are endogenous non‐coding RNAs highly conserved among different species and may act as completing RNAs to bind miRNAs.^18^, ^19^, ^20^ Therefore, we identified miR‐9‐3p as a potential miRNA that may sponge circVMA21 according to bioinformatics analysis and a previous study.^21^ To identify whether circVMA21 and miR‐9‐3p bound directly, a luciferase assay was performed. Cotransfection of circVMA21 and miR‐9‐3p repressed the reporter activity in HK‐2 cells (Figure 4A,B). To verify the correlation between circVMA21 and miR‐9‐3p, we used RIP assay for AGO2 in HK2 cells, and qRT‐PCR data showed that miR‐9‐3p targeted circVMA21 in the AGO2‐dependent manner (Figure 4C). Also, we found that overexpression of circVMA21 could notably reduce the expression level of miR‐9‐3p (Figure 4D). After the confirmation of directly binding between circVMA21 and miR‐9‐3p, we explored the expression level of miR‐9‐3p both in sepsis‐associated AKI patients and LPS‐treated HK‐2 cells and found the miR‐9‐3p levels were all up‐regulated (Figure 4E)

**FIGURE 5:**
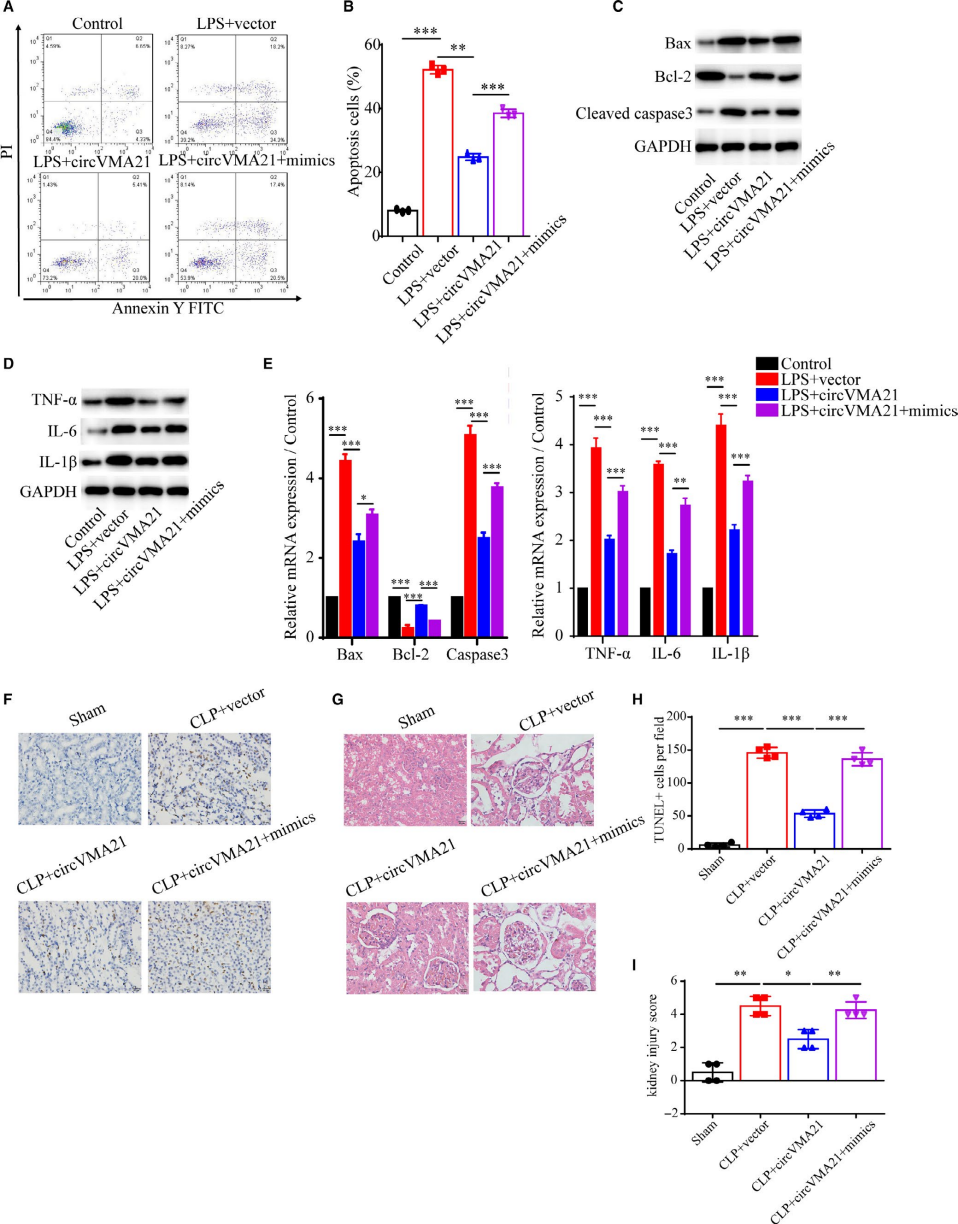
circVMA21 functioned as a sponge of miR‐9‐3p. (A) Apoptosis levels were measured by flow cytometry. Groups: control, LPS + vector, LPS + circVMA21, LPS + circVMA21+mimics infected HK‐2 cells, and (B) statistical result of the apoptosis levels. Levels of (C) apoptosis related proteins, Bax, Bcl‐2 and active Caspase3 and (D) inflammation related proteins, TNF‐α, IL‐6 and IL‐1β, were measured with WB. (E) Relative mRNA expression levels of Bax, Bcl‐2, Caspase3, TNF‐α, IL‐6 and IL‐1β were measured with quantitative PCR. (F) Apoptosis of renal tubular epithelial cells in group Sham, CLP + vector, CLP + circVMA21, CLP + circVMA21+mimic was detected using TUNEL methods. (G) Pathological morphology HE staining of the renal tissue from different groups. (H) Quantification of TUNEL positive apoptosis cells per field. (I) Semi‐quantitative histopathological score of renal tissue. *P < 0.05, ***P < 0.001. Data were presented as mean ± SD from three independent experiments

Therefore, we suggested that circVMA21 could reduce renal apoptosis and inflammation by sponging miR‐9‐3p. First, we examined the apoptosis level of HK‐2 cells by flow cytometry and the expression level of apoptosis related protein, confirmed that gain of function of miR‐9‐3p by using miR‐9‐3p mimics could lead to increased apoptosis and inflammation in HK‐2 cells (Figure 5A‐E). Next, the results of TUNEL assay and HE staining using the kidney samples from CLP‐induced sepsis‐associated AKI rats showed that circVMA21 reduced apoptosis through down‐regulation of miR‐9‐3p, which is consistent with the in *vitro* results (Figure 5F‐I).

### 3.5 circVMA21 reduced apoptosis and inflammation via miR‐9‐3p/SMG1 axis

**FIGURE 6:**
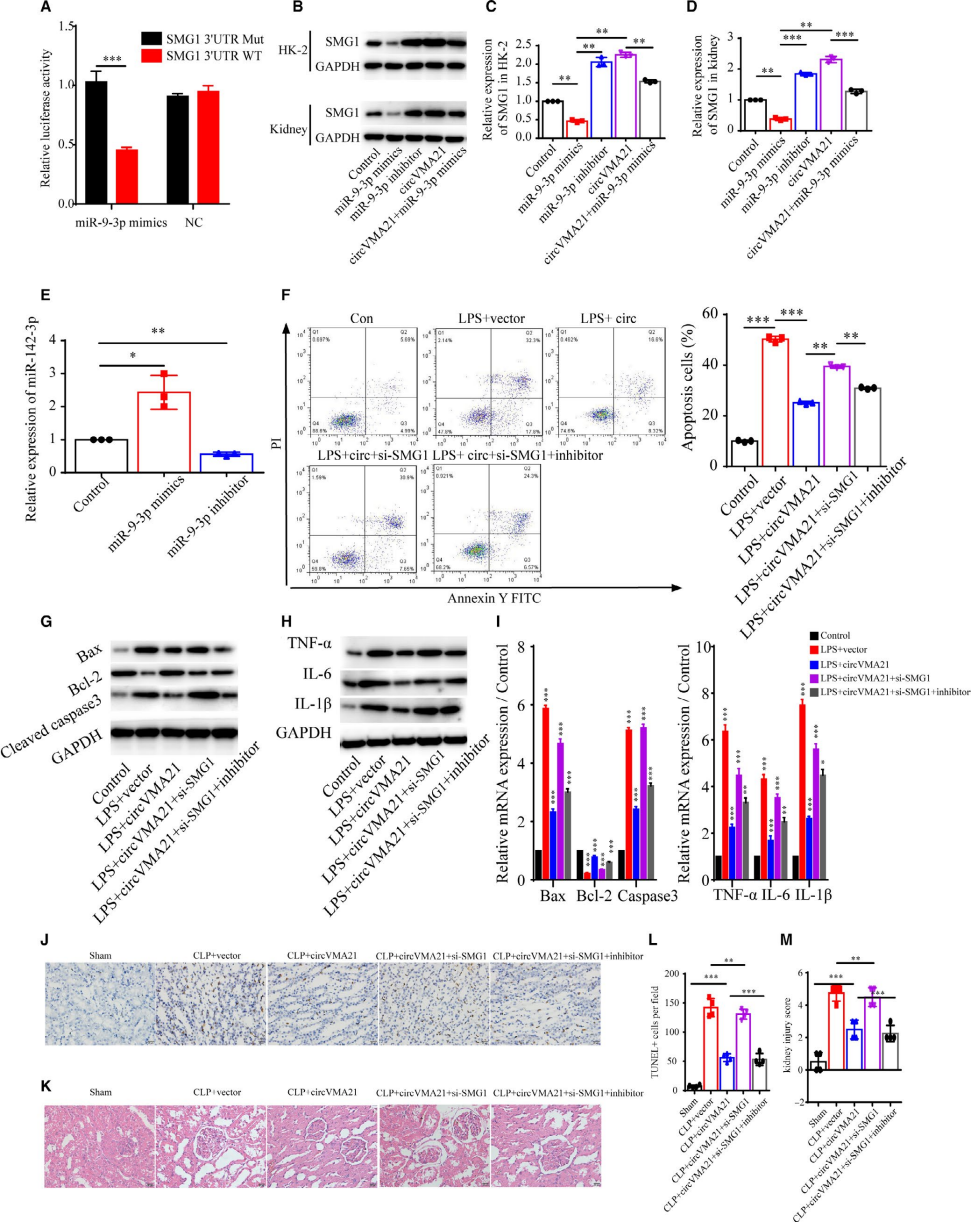
circVMA21 reduced apoptosis and inflammation via miR‐9‐3p/SMG1 axis. (A) Luciferase activity of miR‐9‐3p reporter was measured to confirm direct binding between miR‐9‐3p and SMG1 in HK‐2 cells. (B) Levels of SMG1 and relative expression of SMG1 mRNA (C) in HK‐2 cells and (D) in the rat kidney were measured using WB and quantitative PCR. Groups: normal control, miR‐9‐3p mimics, miR‐9‐3p inhibitor, circVMA21, circVMA21 + miR‐9‐3p mimics. (E) Relative expression level of miR‐142‐3p. (F) Apoptosis levels in Control, LPS + vector, LPS + circVMA21, LPS + circVMA21+si‐SMG1, LPS + circVMA21+si‐SMG1 + inhibitor transfected HK‐2 cells were determined by flow cytometry. Levels of (G) apoptosis related proteins and (H) inflammation related proteins in HK‐2 cells were measured with WB. (I) Relative expression level of Bax, Bcl‐2, Caspase3, TNF‐α, IL‐6 and IL‐1β were measured with quantitative PCR. (J) Apoptosis of renal tubular epithelial cells in Sham, CLP + vector, CLP + circVMA21, CLP + circVMA21+si‐SMG1, CLP + circVMA21+si‐SMG1 + inhibitor, CLP + circVMA21+si‐SMG1 + inhibitor rats were mainly detected by TUNEL method. (K) Pathological morphology HE staining of the renal tissue from different groups. (L) Quantification of TUNEL positive apoptosis cells per field. (M) Semi‐quantitative histopathological score of renal tissue. *P < 0.05, ***P < 0.001. Data were presented as mean ± SD from three independent experiments

Next, for the purpose of searching for the probable effector protein that in circVMA21/miR‐9‐3p axis in HK‐2 cells, we conducted the TargetScan analysis (TargetScan Release 7.1) and revealed that the 3′‐UTR of SMG1, a potential tumour suppressor in several different cancers, contained a miR‐9‐3p binding site.^22^ Then, the luciferase reporter assay showed that miR‐9‐3p could significantly reduce the luciferase activity, indicating the specific binding between miR‐9‐3p and SMG1 mRNA (Figure 6A). We then found that up‐regulation of miR‐9‐3p could notably suppress the expression of SMG1, and down‐regulation of miR‐9‐3p with inhibitor increased the SMG1 level, indicating circVMA21 functioned via miR‐9‐3p/SMG1 axis (Figure 6B‐D). In AKI patients, miR‐9‐3p was up‐regulated and bound to the mRNA of SMG1, leading to the change of network of downstream signal pathways. MiR‐142‐3p was negatively regulated by TUG1, and up‐regulation of TUG1 could relieve the injury induced by LPS.^23^ We found that up‐regulation of miR‐9‐3p increased the expression level of miR‐142‐3p (Figure 6E). For further confirmation of the role of SMG1 in the signalling pathway, we explored the apoptosis phenotype in the LPS‐induced HK‐2 cell model of sepsis‐associated AKI and found down‐regulation of SMG1 while overexpression of circVMA21 reversed the alleviated apoptosis and inflammation by circVMA21 (Figure 6F‐I). With the application of TUNEL and HE staining using the kidney tissue sampled from the sepsis‐associated AKI rat model, we confirmed that, in vivo, down‐regulation of SMG1 also led to increased levels of apoptosis and inflammation, and inhibition of miR‐9‐3p then led to alleviated phenotype (Figure 6J‐M). Combining both in vitro and in vivo results, we could safely conclude that SMG1 mRNA is the target of miR‐9‐3p and it can be regulated by circVMA21.

## 4 DISCUSSION

Exploration of the functions of circular RNAs expressed in the kidney may provide clues and information for the therapeutic targets exploitation. In our study, we found the overexpression of circVMA21 in the kidney could alleviate sepsis‐associated AKI by evaluating several sepsis‐associated AKI biomarkers and the kidney injury score in rats. Furthermore, we found that up‐regulation of circVMA21 reduced apoptosis and inflammation via miR‐9‐3p/SMG1 axis. Also, overexpression of circVMA21 decreased oxidative stress in sepsis‐associated AKI.

CLP has been used to produce severe sepsis and then AKI demonstrated by a greater decrease in kidney mircocirculation.^24^ Also, LPS has been used to mimic the sepsis‐associated AKI symptoms in HK‐2 cells.^25^ Here, by evaluating several parameters of sepsis‐associated AKI in the CLP‐induced rat and LPS‐induced cell models, we demonstrated that circVMA21 reduced apoptosis and inflammation both in *vitro* and in *vivo*.

Circular RNAs, which are generally expressed specifically in cell types, can serve as a sponge of miRNA and then modulate gene expression and protein synthesis.^26^ Bioinformatics analysis revealed that potential binding site of miR‐9‐3p were exist in circVMA21. To test whether miR‐9‐3p directly bound to circVMA21, we performed luciferase and RIP assay, and data confirmed the direct binding. MiR‐9‐3p, the minor product of miR‐9, is able to suppress the proliferation of hepatocellular carcinoma cells via targeting heparin‐binding growth factor 5 and regulate the proliferation and apoptosis of ovarian cancer cells through targeting the extracellular signal‐regulated protein kinase 1 and 2.^27^, ^28^ Here, to investigate the role of miR‐9‐3p, we employed miR‐9‐3p mimics for the gain of function study and found that miR‐9‐3p mimics led to increased apoptosis and inflammation.

MiRNAs can bind to the 3′‐UTR of target mRNAs resulting in the mRNA degradation and inhibition of the mRNA protein translation.^29^, ^30^ In this study, we screened for the candidate effector proteins using TargetScan analysis and suggested the 3′‐UTR of SMG1 mRNA contained potential binding sites for miR‐9‐3p. Then, we confirmed the direct binding of miR‐9‐3p and SMG1 mRNA using the luciferase assay and that the expression level of SMG1 was indeed negatively correlated with miR‐9‐3p in HK‐2 cells. SMG1, a member of the phosphoinositide kinase‐like kinase family, plays a role in several diseases progression, including pancreatic cancer, *Physcomitrella* patens and inflammation‐enhanced cancer.^31^, ^32^, ^33^ SMG1 is involved multiple signal pathways, such as oxidative stress and TNF‐induced apoptosis.^34^ As human SMG1 was highly expressed in kidney,^35^ we hypothesis SMG1 plays an important role in the sepsis‐associated acute kidney injury, which could also lead to excessive production of ROS and cell apoptosis. In vitro and in vivo data then confirmed that down‐regulation of SMG1 resulted in increased levels of apoptosis and inflammation, and the phenotypes could be reversed by the inhibition of miR‐9‐3p.

As SMG1 functioned in oxidative stress resistance during tumour formation and inflammation, we then attempted to whether cicrVMA21 played a role in the resistance of oxidative stress.^32^ We measured several parameters of oxidative stress, including levels of ROS, MDA, GSH and the activities of SOD and CAT, and found the oxidative stress caused by sepsis‐associated AKI was reduced by up‐regulation of circVMA21.

In conclusion, circVMA21 could alleviate sepsis‐associated AKI through reduced the levels of apoptosis, inflammation and oxidative stress. Additionally, we confirmed that circVMA21 directly bound with miR‐9‐3p to regulate the synthesis of SMG1. However, the CLP‐induced AKI rats model cannot perfectly mimic the phenotypes of human sepsis‐associated acute kidney injury, and more information should be analysed using human samples from patients. And the pathology of sepsis‐associated acute kidney injury is related to multiple signal pathways. The network of molecules correlated with circVMA21 should be further explored. Our results suggested that circVMA21 could be a hopeful therapeutic target for the drug development of sepsis‐associated AKI.

## CONFLICT OF INTEREST

None.

## AUTHOR CONTRIBUTION

**Chuanfu Sun:** Formal analysis (equal); Investigation (equal); Methodology (equal); Software (equal); Validation (equal); Visualization (equal); Writing‐original draft (supporting); Writing‐review & editing (supporting). **Yan Shi:** Formal analysis (equal); Investigation (equal); Methodology (equal); Software (equal); Validation (equal); Visualization (equal); Writing‐original draft (supporting); Writing‐review & editing (supporting). **Wenhan Ge:** Formal analysis (supporting); Investigation (supporting); Methodology (supporting); Software (supporting); Validation (supporting); Writing‐original draft (supporting); Writing‐review & editing (supporting). **Yeping Du:** Formal analysis (supporting); Investigation (supporting); Methodology (supporting); Software (supporting); Validation (supporting); Visualization (supporting); Writing‐original draft (supporting); Writing‐review & editing (supporting). **Nan‐Bin Hu:** Conceptualization (lead); Data curation (lead); Funding acquisition (lead); Project administration (lead); Resources (lead); Supervision (lead); Writing‐original draft (lead); Writing‐review & editing (lead).

## Data Availability

Data in this research for supporting the results are all included within the article.

## References

[1] Poston JT , Koyner JL . Sepsis associated acute kidney injury. BMJ. 2019;364:k4891. 30626586 10.1136/bmj.k4891 PMC6890472

[2] Bagshaw SM , Lapinsky S , Dial S , et al. Cooperative Antimicrobial Therapy of Septic Shock Database Research G. Acute kidney injury in septic shock: clinical outcomes and impact of duration of hypotension prior to initiation of antimicrobial therapy. Intensive Care Med. 2009;35:871‐881. 19066848 10.1007/s00134-008-1367-2

[3] Hoste EA , Lameire NH , Vanholder RC , Benoit DD , Decruyenaere JM , Colardyn FA . Acute renal failure in patients with sepsis in a surgical ICU: predictive factors, incidence, comorbidity, and outcome. J Am Soc Nephrol. 2003;14:1022‐1030. 12660337 10.1097/01.asn.0000059863.48590.e9

[4] Oppert M , Engel C , Brunkhorst FM , et al. German Competence Network S. Acute renal failure in patients with severe sepsis and septic shock–a significant independent risk factor for mortality: results from the German Prevalence Study. Nephrol Dial Transplant. 2008;23:904‐909. 18065435 10.1093/ndt/gfm610

[5] Rangel‐Frausto MS , Pittet D , Costigan M , Hwang T , Davis CS , Wenzel RP . The natural history of the systemic inflammatory response syndrome (SIRS). A prospective study. JAMA. 1995;273:117‐123. 7799491

[6] Brandenburger T , Salgado Somoza A , Devaux Y , Lorenzen JM . Noncoding RNAs in acute kidney injury. Kidney Int. 2018;94:870‐881. 30348304 10.1016/j.kint.2018.06.033

[7] Jin J , Sun H , Shi C , et al. Circular RNA in renal diseases. J Cell Mol Med. 2020;24:6523‐6533. 32333642 10.1111/jcmm.15295 PMC7299708

[8] Liu Z , Wang Y , Shu S , Cai J , Tang C , Dong Z . Non‐coding RNAs in kidney injury and repair. Am J Physiol Cell Physiol. 2019;317:C177‐C188. 30969781 10.1152/ajpcell.00048.2019

[9] Zhou J , Chen H , Fan Y . Systematic analysis of the expression profile of non‐coding RNAs involved in ischemia/reperfusion‐induced acute kidney injury in mice using RNA sequencing. Oncotarget. 2017;8:100196‐100215. 29245971 10.18632/oncotarget.22130 PMC5725013

[10] Cheng XF , Zhang L , Zhang K , et al. Circular RNA VMA21 protects against intervertebral disc degeneration through targeting miR‐200c and X linked inhibitor‐of‐apoptosis protein. Ann Rheum Dis. 2018;77:770‐779. 29343508 10.1136/annrheumdis-2017-212056 PMC5909753

[11] Yang P , Gao R , Zhou W , Han A . Protective impacts of circular RNA VMA21 on lipopolysaccharide‐engendered WI‐38 cells injury via mediating microRNA‐142‐3p. BioFactors. 2019;46(3):381–390. 31793712 10.1002/biof.1593

[12] Brooks HF , Osabutey CK , Moss RF , Andrews PL , Davies DC . Caecal ligation and puncture in the rat mimics the pathophysiological changes in human sepsis and causes multi‐organ dysfunction. Metab Brain Dis. 2007;22:353‐373. 17828620 10.1007/s11011-007-9058-1

[13] Huang Y , Zhou F , Shen C , Wang H , Xiao Y . LBP reduces theinflammatory injuryof kidney in septic rat and regulates the Keap1‐Nrf2ARE signaling pathway1. Acta Cir Bras. 2019;34:e20190010000003. 30785504 10.1590/s0102-865020190010000003 PMC6585919

[14] Aebi H . Catalase in vitro. Methods Enzymol. 1984;105:121‐126. 6727660 10.1016/s0076-6879(84)05016-3

[15] Forbes JM , Coughlan MT , Cooper ME . Oxidative stress as a major culprit in kidney disease in diabetes. Diabetes. 2008;57:1446‐1454. 18511445 10.2337/db08-0057

[16] Wang JN , Yang Q , Yang C , et al. Smad3 promotes AKI sensitivity in diabetic mice via interaction with p53 and induction of NOX4‐dependent ROS production. Redox Biol. 2020;32:101479. 32143149 10.1016/j.redox.2020.101479 PMC7058410

[17] Hosohata K . Role of oxidative stress in drug‐induced kidney injury. Int J Mol Sci. 2016;17:1826. 10.3390/ijms17111826 PMC5133827 27809280

[18] Yu CY , Li TC , Wu YY , et al. The circular RNA circBIRC6 participates in the molecular circuitry controlling human pluripotency. Nat Commun. 2017;8:1149. 29074849 10.1038/s41467-017-01216-w PMC5658440

[19] Guo Y , Luo F , Liu Q , Xu D . Regulatory non‐coding RNAs in acute myocardial infarction. J Cell Mol Med. 2017;21:1013‐1023. 27878945 10.1111/jcmm.13032 PMC5387171

[20] Meng X , Li X , Zhang P , Wang J , Zhou Y , Chen M . Circular RNA: an emerging key player in RNA world. Brief Bioinform. 2017;18:547‐557. 27255916 10.1093/bib/bbw045

[21] Wu J , Li DD , Li JY , et al. Identification of microRNA‐mRNA networks involved in cisplatin‐induced renal tubular epithelial cells injury. Eur J Pharmacol. 2019;851:1‐12. 30768982 10.1016/j.ejphar.2019.02.015

[22] Du Y , Lu F , Li P , et al. SMG1 acts as a novel potential tumor suppressor with epigenetic inactivation in acute myeloid leukemia. Int J Mol Sci. 2014;15:17065‐17076. 25257528 10.3390/ijms150917065 PMC4200422

[23] Liu X , Hong C , Wu S , et al. Downregulation of lncRNA TUG1 contributes to the development of sepsis‐associated acute kidney injury via regulating miR‐142‐3p/sirtuin 1 axis and modulating NF‐kappaB pathway. J Cell Biochem. 2019. 10.1002/jcb.28409 30834562

[24] Seely KA , Holthoff JH , Burns ST , et al. Hemodynamic changes in the kidney in a pediatric rat model of sepsis‐induced acute kidney injury. Am J Physiol Renal Physiol. 2011;301:F209‐F217. 21511700 10.1152/ajprenal.00687.2010 PMC3129882

[25] Zhang D , Li Y , Liu Y , Xiang X , Dong Z . Paclitaxel ameliorates lipopolysaccharide‐induced kidney injury by binding myeloid differentiation protein‐2 to block Toll‐like receptor 4‐mediated nuclear factor‐kappaB activation and cytokine production. J Pharmacol Exp Ther. 2013;345:69‐75. 23318472 10.1124/jpet.112.202481 PMC3608443

[26] Li X , Yang L , Chen LL . The biogenesis, functions, and challenges of circular RNAs. Mol Cell. 2018;71:428‐442. 30057200 10.1016/j.molcel.2018.06.034

[27] Kornmann M , Ishiwata T , Beger HG , Korc M . Fibroblast growth factor‐5 stimulates mitogenic signaling and is overexpressed in human pancreatic cancer: evidence for autocrine and paracrine actions. Oncogene. 1997;15:1417‐1424. 9333017 10.1038/sj.onc.1201307

[28] Liu X , Li G . MicroRNA‐133b inhibits proliferation and invasion of ovarian cancer cells through Akt and Erk1/2 inactivation by targeting epidermal growth factor receptor. Int J Clin Exp Pathol. 2015;8:10605‐10614. 26617770 PMC4637585

[29] Wang QX , Zhu YQ , Zhang H , Xiao J . Altered MiRNA expression in gastric cancer: a systematic review and meta‐analysis. Cell Physiol Biochem. 2015;35:933‐944. 25633747 10.1159/000369750

[30] Lv J , Xia K , Xu P , et al. miRNA expression patterns in chemoresistant breast cancer tissues. Biomed Pharmacother. 2014;68:935‐942. 25451164 10.1016/j.biopha.2014.09.011

[31] Lloyd JPB , Lang D , Zimmer AD , Causier B , Reski R , Davies B . The loss of SMG1 causes defects in quality control pathways in *Physcomitrella patens* . Nucleic Acids Res. 2018;46:5822‐5836. 29596649 10.1093/nar/gky225 PMC6009662

[32] Roberts TL , Ho U , Luff J , et al. Smg1 haploinsufficiency predisposes to tumor formation and inflammation. Proc Natl Acad Sci USA. 2013;110:E285‐E294. 23277562 10.1073/pnas.1215696110 PMC3557096

[33] Wong C , Chen F , Alirezaie N , et al. A region‐based gene association study combined with a leave‐one‐out sensitivity analysis identifies SMG1 as a pancreatic cancer susceptibility gene. PLoS Genet. 2019;15(8):e1008344– 10.1371/journal.pgen.1008344 31469826 PMC6742418

[34] Yamashita A , Izumi N , Kashima I , et al. SMG‐8 and SMG‐9, two novel subunits of the SMG‐1 complex, regulate remodeling of the mRNA surveillance complex during nonsense‐mediated mRNA decay. Genes Dev. 2009;23:1091‐1105. 19417104 10.1101/gad.1767209 PMC2682953

[35] Denning G , Jamieson L , Maquat LE , Thompson EA , Fields AP . Cloning of a novel phosphatidylinositol kinase‐related kinase: characterization of the human SMG‐1 RNA surveillance protein. J Biol Chem. 2001;276:22709‐22714. 11331269 10.1074/jbc.C100144200

